# Methicillin-resistant *Staphylococcus aureus* mupirocin resistance rates in a large healthcare system

**DOI:** 10.1017/ash.2023.343

**Published:** 2023-09-29

**Authors:** Mindy Sampson, Robert Fairman, Elizabeth Palavecino, Werner Bischoff, Julie Williamson, Shelley Keste, Catherine Passaretti

## Abstract

Methicillin-resistant *Staphylococcus aureus* (MRSA) is a common etiology of hospital-acquired infections (HAIs). One strategy to reduce HAIs due to MRSA involves a multistep decolonization process. This often involves nasal application of mupirocin 2% ointment. In our institution, when individuals meet criteria for decolonization, we recommend 5 days of treatment given twice daily. High levels of mupirocin resistance have been reported in some hospital systems, with >80% of tested isolates being resistant. To better understand our resistance levels, we selected 238 MRSA isolates from blood cultures to be tested for mupirocin resistance to correlate the presence of resistance and use of mupirocin for decolonization. We choose to assess MRSA blood isolates rather than nasal swabs given that we aim to prevent invasive MRSA infections, including blood stream infections, with decolonization. The blood cultures were collected from 11 acute-care facilities within our system from March 2021 through June 2022. High-level resistance was defined as an MIC >1,024 μg/mL according to Clinical and Laboratory Standards Institute guidelines. Of those, 7.14% showed high level resistance, and 76.47% occurred in those who were exposed to mupirocin and 23.53% occurred in those without mupirocin exposure (*P* = .0094). On average, those with high-level resistance had had more recent exposure to mupirocin compared to those without resistance, which was statistically significant. Also, those with high resistance, on average, received more doses of mupirocin, although this was not statistically significant. **Conclusions:** More recent and higher number of doses of mupirocin were associated with the development of resistance, which is consistent with what we know from pharmacodynamics of antibiotic resistance with other agents. These findings may be particularly important for those patients who have frequent hospitalizations and often require decolonization. Understanding baseline mupirocin resistance levels in an institution can assist with determining decolonization strategies.

**Figure:**
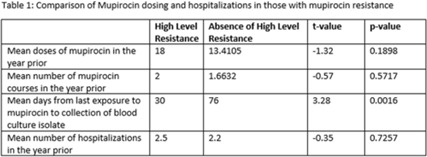

**Disclosures:** None

